# Online Survey Retention and Re-engagement: Learning from the COVID-19 Social Study

**DOI:** 10.1177/1525822X241289870

**Published:** 2025-01-08

**Authors:** Feifei Bu, Alexandru Cernat, Andrew Steptoe, Daisy Fancourt

**Affiliations:** 1 Department of Behavioural Science and Health, 4919 University College London, London, UK; 2 Department of Social Statistics, 5292 University of Manchester, Manchester, UK

## Abstract

This article examines factors associated with survey attrition/retention in an online panel survey with weekly/monthly follow-ups during the COVID-19 pandemic. It also explores the effectiveness of making contact with dropout participants and factors associated with sample re-engagement, as well as motivations and barriers to maintaining survey participation. Our data consisted of over 1.2 million records from over 70,000 participants collected between March 2020 and April 2022. On average, 92.7% participants during weekly follow-ups and 95.9% during monthly follow-ups participated again in a later wave. The high retention rates, to some extent, could be attributed to a high level of altruistic motivations during a global health crisis and to the use of retention strategies to create a project community. A similar set of factors were related to both survey attrition/retention and re-engagement. However, some differences were also found, indicating the possibility of distinct decision processes.

## Introduction

On March 11, 2020, the World Health Organization (WHO) declared COVID-19 a pandemic. This global health crisis created an urgent demand for rapid scientific research. The University College London (UCL) COVID-19 Social Study (CSS) was launched to provide a granular detail of how people were affected day-by-day and week-by-week during the pandemic. Between March 2020 and April 2022, the study collected over 1.2 million records from over 70,000 participants. CSS has become a major source of knowledge about psychosocial experiences during COVID-19, leading to over 100 scientific papers to date. Moreover, it is also an important case study in the design and conduct of rapid online panel studies. CSS recruited a high volume of participants in the short space of just a few weeks. Despite involving regular, intensive follow-ups, retention rates for the study remained high (Fancourt et al. 2022). CSS provides an opportunity to explore a number of questions that are of core relevance to scientists designing or leading online panel studies.

First, what are the attrition/retention rates in a high-frequency online panel study? Online studies like CSS are known to be subject to higher attrition rates compared to other survey modes, such as face-to-face interviews (Manfreda et al. 2008). Although there is a rich literature on panel attrition broadly (e.g., Behr et al. 2005; Lugtig 2014), there is a lack of evidence on online panels, with few exceptions (Maslovskaya and Lugtig 2022; Yu et al. 2022). CSS comprised both weekly and monthly phases, allowing for direct comparisons of attrition/retention rates between different survey frequencies. It also included “re-engagement” waves, where people who had been lost to follow-up were recontacted and invited to rejoin the study. It, therefore, provides a valuable case study for understanding the success of different approaches.

Second, what factors predict attrition and other related measures? This is key to assessing attrition biases. Previous research has shown that attrition can be affected by societal-level factors, survey design and participant characteristics (Groves and Couper 2012; Watson and Wooden 2009). Societal-level factors include, for example, the felt-legitimacy of organizations conducting the survey and social cohesion in the society, which may affect an individual’s attitude and behavior (Groves and Couper 2012). Relevant survey design attributes include incentives, the number of contacts, follow-up frequency, questionnaire length, and survey topic (Frankel and Hillygus 2014; Keusch 2015). Individual characteristics include sex, age, race/ethnicity, education, employment status, income, household composition, area of living, health and so forth (Beller et al. 2022; Bhamra et al. 2008; Uhrig 2008).

Third, what are the motivations and barriers for participating in online panel studies? Previous work has identified dozens of different strategies that can affect retention in panel studies (Teague et al. 2018). However, few studies involve as many waves of data collection as CSS. Further, CSS provided an opportunity to test whether particular design and contextual factors influenced participation. The unique social context of COVID-19 could have led to an increased sense of social responsibility, which might have influenced their participation in COVID-19 related surveys (Groves and Couper 2012). But by the same token, participants might have experienced a different set of or heightened challenges, such as time, COVID-19-related adversities, and stresses.

The present study had four research aims: (1) to map the retention/attrition rates of CSS over time; (2) to identify predictors of attrition; (3) to explore predictors of re-engagement when participants rejoined the study; and (4) to understand motivations and barriers to participation. The findings are valuable not only for CSS data users to properly correct for potential biases, but also for adding to the existing literature on attrition in online panels and informing future survey designs on how to optimally recruit and retain participants.

## Method

### Study Recruitment

CSS had a three-fold recruitment strategy that was designed to maximize the heterogeneity of the sample and to ensure coverage of vulnerable and marginalized groups. First, convenience sampling was used, including promoting the study through existing networks and mailing lists, print and digital media coverage, and social media. This included advertising the study through databases of adults who had previously consented to be involved in health research (such as UCL BioResource and HealthWise Wales) and through the UKRI Mental Health Research Networks. Second, more targeted recruitment was undertaken focusing on (1) individuals from a low-income background; (2) individuals with no or few educational qualifications; and (3) individuals who were unemployed. This was achieved through partnership work with targeted advertising companies and recruitment companies who provided pro-bono support for the study, including Find Out Now, SEO Works, FieldworkHub, and Optimal Workshop. Third, the study was promoted via partnerships with third sector organizations to vulnerable groups, including adults with pre-existing mental health conditions, older adults, carers, and people experiencing domestic violence or abuse. This included utilizing partnerships from the 1,500-strong membership of the UKRI MARCH Mental Health Research Network (https://www.marchlegacy.org/).

### Study Design

Participants provided an email address on first enrollment and completed the baseline questionnaire. They were then recontacted via the email provided for follow-up questionnaires. At each follow-up, a daily reminder was sent out automatically up to two times for those who did not respond to the initial invite. If participants did not complete the survey following the two reminders, they would stop receiving future invitations. There was no monetary incentive for taking part in the study.

**Figure 1.**
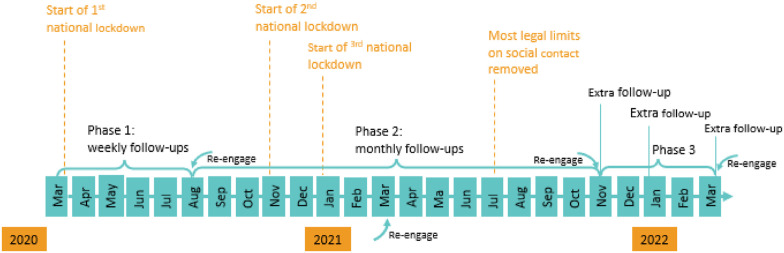
The timeline of the COVID-19 Social Study surveys.

Figure 1 shows the study timeline and how it is related to the three national lockdowns in the United Kingdom. There were three phases of data collection. The study commenced on March 21, 2020, with participants receiving follow-up questionnaires seven days after their last completion until August 2020 (phase 1). From then, participants were followed up monthly until November 2021 (phase 2). At the start of phase 2, to have a spread of responses across weeks, participants were randomized into four groups to receive their first monthly invitation in week 1, 2, 3, or 4 beginning August 24, 2020. Follow-up questionnaires were sent out 28 days after their last completion (Figure S1). In the third and final phase, additional irregularly follow-ups were carried out in November 2021 and January and March 2022.

To address loss to follow-up, we re-approached all participants who had stopped completing questionnaires but had not formally unsubscribed from the study to rejoin at periodic intervals across the study. These dates were August–September 2020 (start of phase 2), March 2021 (phase 2), November 2021 (start of phase 3), and, finally, March 2022 (Figure 1). The study was approved by the UCL Research Ethics Committee [12467/005], and all participants gave written informed consent.

### Participant Characteristics

In total, 73,222 participants took part in the survey. In the sample, 81.7% of participants were living in England, 10.6% in Wales, 6.6% in Scotland and 1.2% in Northern Ireland (Table S1). There was an overrepresentation of women (74.9%), people with a degree or above (66.7%) and an underrepresentation of young adults under 30 (10.9%) and people with an ethnic minority background (6.1%). However, these imbalances between the sample and targeted population can be corrected by weighting in data analysis (Table S1).

### Measurement and Analysis

To map retention rates, descriptive analyses were conducted to assess changes in sample size and retention rate over time across different study phases. Retention rate was defined as the percentage of participants having responded at least once to any of the subsequent surveys. Further, we also adopted a more restricted definition of retention—the percentage of participants having responded to the next consecutive follow-up survey.

To identify predictors of attrition, Cox regression models were fitted to examine factors that were associated with the length of time from study entry to dropout or the end of study periods (Machin et al. 2006). For simplicity, we focused on the first phase of the study when participants were followed-up weekly (before reinviting dropouts). Entry time was the date of first enrolling for each participant and dropout time was defined as nine days after the last response in phase 1—participants did not respond to the next weekly follow-up after two daily reminders. Participants who took part in August 2020 were considered as censoring without attrition. We considered a range of potential predictors as shown in Table S1. All predictors were measured at baseline. In the same model, we also included the day of week (Sunday to Saturday) when participants received the survey invite before they dropped out.

Re-engagement was defined as people who dropped out previously re-joined the study when re-approached. We looked at the start of phase 2 (August–September 2020) when the study was converted from weekly to monthly follow-ups. In these analyses, we were able to consider noncontact, for example, due to typographical errors in email addresses, or inbox being full, by excluding email addresses that were undeliverable. Re-engagements were analyzed using logistic regression models, including the same set of predictors as the Cox regression model, except that the day of week was replaced by study groups (1, 2, 3, 4), which participants were randomly assigned to at the start of phase 2. Participants from the same group received the survey invite on the same day for their first phase 2 survey.

Finally, to understand motivations and barriers, the last survey in November 2022 included two open-ended questions: (1) What has motivated you to continue taking part over the past two years? (2) What have you found challenging about participating in the study or is there anything we could improve if a similar study is run in the future? We conducted descriptive analyses on these free text data to understand people’s motivations and challenges. For this analysis, we restricted the sample to those who responded to each of these questions, excluding participants having reported no motivation or challenge. Text data were pre-processed using an iterative process where we removed common stop words (e.g., the, I, and, etc.) and reduced words to their linguistic stem (e.g., interest, interesting, interests, interested). Text analysis was mainly performed using unigrams (single words), but we included compound words with high level of co-occurrence (minimum frequency of 20). Main analyses were unweighted, but sensitivity analyses were carried out after using entropy balancing weights, accounting for country, age, gender, ethnicity and education. All analyses were carried out using Stata V17, except for free text analyses that were conducted in R 4.3.1.

## Results

### What Were the Rates of Study Retention across the Two Years?

**Figure 2.**
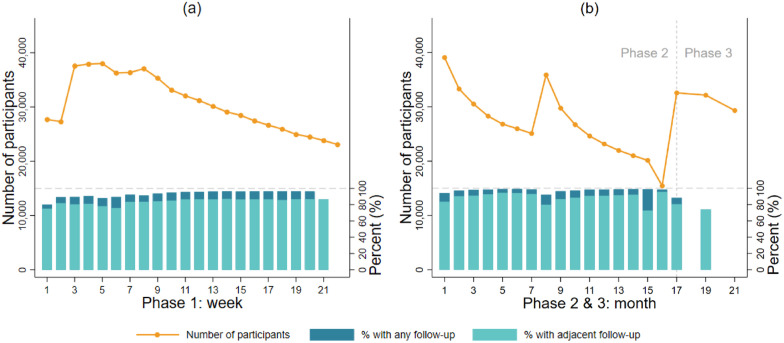
Sample sizes and retention rates across weeks/months in different phases of the COVID-19 Social Study.

Figure 2(a) shows the number of participants and retention rates in each week during the first phase of the study (Table S2). The number of participants increased in the first few weeks because we were actively recruiting until the end of May 2020. More than 23,000 unique participants took part in the study each week, and over 38,000 at peak. The retention rates during phase 1 were 92.7% on average (83.7% for consecutive retention).

Figure 2(b) shows the number of participants and retention rates across months during phase 2 and 3 (Table S3). The numbers of participants increased each time participants were re-invited, and then decreased over time. This pattern was the same across the four subgroups that participants were randomly allocated to (Figure S2). Consistently, the retention rate in each month remained high (95.9% on average, 87.8% consecutive retention), except for a noticeable decrease at the end of phase 2 (month 15-16).

### What Factors Predicted Study Attrition?

**Figure 3.**
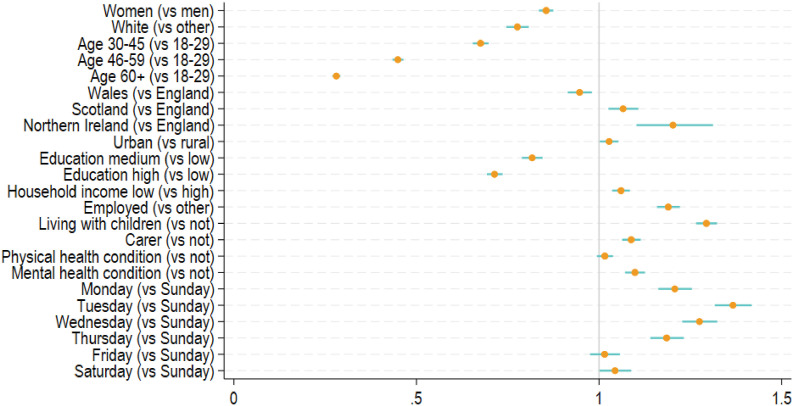
Predictors of study attrition: hazard ratios (HRs) and 95% confidence intervals from Cox regression models (*N* = 62,772, unweighted).

Figure 3 reports factors that were related to attrition at phase 1 between March and August 2020 (see also Table S4). Women (Hazard Ratio (HR) = 0.85, 95% CI = 0.84–0.87), people of white–ethnicity (HR = 0.78, 95% CI = 0.75–0.81), middle-aged and older adults (HR = 0.28–0.68), people living in Wales (HR = 0.95, 95% CI = 0.9–0.98) and the better educated (HR = 0.71–0.82) had a lower attrition hazard. Urban residents (HR = 1.03, 95% CI = 1.00–1.05), people living in Scotland or Northern Ireland (HR = 1.07–1.20), those from low-income households (HR = 1.06, 95% CI = 1.041.08), those in employment (HR = 1.19, 95% CI = 1.16–1.22), people living with children (HR = 1.29, 95% CI = 1.27–1.32) and carers (HR = 1.09, 95% CI = 1.06–1.11) had a higher attrition hazard. We found no evidence that physical health was related to attrition, but people with poor mental health were 10% more likely to drop out (HR = 1.10, 95% CI = 1.07–1.13). Compared to Sunday, people who received the last invite from Monday to Thursday had a higher attrition hazard (HR = 1.18–1.37). There was little difference for Friday and Saturday compared to Sunday. The weighted results were consistent with the unweighted main analyses, except that the difference between rural and urban residents, and the difference between Saturday and Sunday were statistically significant (Table S4).

### Who Re-engaged with the Study when Re-invited?

**Figure 4.**
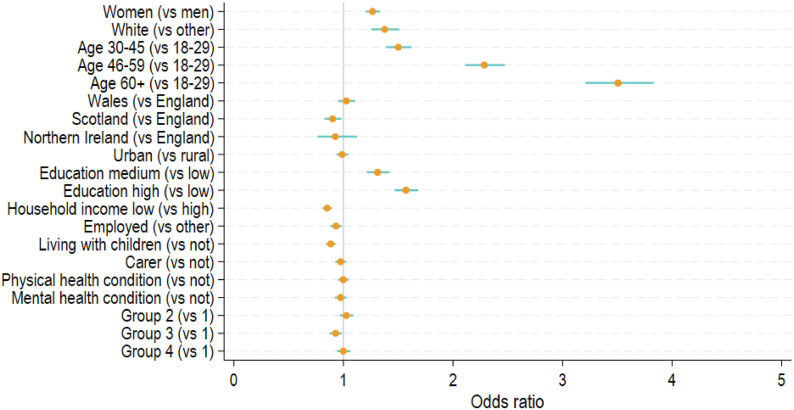
Predictors of re-engagement in the study: odds ratios (ORs) and 95% confidence intervals from logistic regression models for (a) Phase 2 (August 2020, *N* = 39,088, unweighted), and (b) Phase 3 (November 2021, *N* = 41,626, unweighted).

Among those who dropped out the study at phase 1 (*N* = 39,888), about 2% of participants had undeliverable email addresses who were excluded from the analysis. Among the rest (*N* = 39,088), 32.4% participants re-joined at the start of phase 2. Figure 4 shows the factors that were related to re-engagement at the start of phase 2 (August 2020, see also Table S5). Women (Odds Ratio (OR) = 1.27, 95% CI = 1.20–1.33), people of white ethnicity (OR = 1.38, 95% CI = 1.26–1.51), middle-aged and older adults (OR = 1.50–3.51), and the better educated (OR = 1.31–1.57) were more likely to rejoin. People living in Scotland (OR = 0.90, 95% CI = 0.83–0.98), those from low-income households (OR = 0.85, 95% CI = 0.81–0.90), the employed (OR = 0.93, 95% CI = 0.88–0.99) and those living with children (OR = 0.88, 95% CI = 0.84–0.93) had lower odds of rejoining. We found no evidence that carer, physical or mental health conditions were related to re-engagement. Compared to the group who received the survey first, the third group who received it two weeks later had 7% lower odds of rejoining (OR = 0.93, 95% CI = 0.87–0.99). The weighted results were largely similar, except that the country difference between Scotland and England was insignificant (Table S5).

### What Were the Motivations or Barriers for Participants to Stay in the Study?

There were 21,111 participants with valid information on motivation, and 8,887 on challenges. Figure S3a in the Supplement shows the top 20 features in the free-text data on motivations. The most common motivations were altruistic, including base words related to helping (“help”) and making a contribution (“contribut”). The next set of features were related to the importance (“import”) and usefulness (“use”) of the study and its findings to “understand” the impact of pandemic and to “inform” policies/decisions and future (“futur”) plans and actions. This was followed by motivations specifically related to the COVID-19 pandemic and the unprecedented and difficult “time” it created. But it is important to note that time was also used to refer to time availability (“had time,” “takes very little time”). Another common motivation was “interest,” a base form for words such as interested (“in research,” “in the impact of COVID-19”) and interesting (“interest subject of research,” “interesting to take part”). There were also participants who were motivated by a sense of continuity (“wanted to continue what I started”).

As shown in Figure S3b, the top challenge was related to survey design (“question”), for example the length of the questionnaire (“lots of questions,” “long survey”) and repeated measures over time (“repetitive questions,” “same questions”). The second top challenge was time, which mostly referred to time availability. But some participants also commented on the time when the survey invite arrived. If they were not available, they would need to remember to respond in a later time. Moreover, “mental health” was one of the top features, as people found it challenging to think about their own mental health. Another common feature was “covid.” Although some participants did mention losses, stresses, and difficulties because of COVID-19, it was mostly used to describe the context for specific challenges without referring to any challenges related to COVID-19.

## Discussion

### Study Retention and Re-engagement

Overall, the retention rates in the CSS were generally high despite being online, with frequent follow-ups and no monetary incentive. On average, 92.7% of participants during weekly follow-ups and 95.9% during monthly follow-ups participated again in a later wave. It is notable that the retention rates were relatively higher during monthly compared to weekly follow-up phase (despite the initial public interest in engaging in COVID-related studies declining after the first lockdown), suggesting that lower frequency participation might be optimal for sample retention. Our attempts to re-engage study dropouts were considerably successful. More than a third of people who previously dropped out, rejoined the study in August/September 2020, and 26.9 % in November 2021. The re-engagement rates are similar to those in pre-pandemic panel studies (Song et al. 2021; Watson and Wooden 2014).

One plausible explanation for the high retention and strong re-engagement rates is that the study was conducted in the unique context of a global health crisis. This is supported by the free-text data on motivations. The most common features (e.g., help, contribute) are those related to altruism, which is a well-known important factor in why people participate in scientific research (Beskow et al. 2011; Chin et al. 2016). However, it is debatable how big a role altruism plays relative to other factors. For example, previous studies found that altruism plays only a small part compared to reciprocity or reward (Hunter et al. 2012; Mein et al. 2012).

This inconsistency may reflect the impact of the wider context on survey participation. It is possible that COVID-19 sparked a greater need and sense of collective responsibility, which makes altruism the primary motivation for taking part in epidemiological research during the pandemic. This is consistent with our findings that motivations related to the importance of research and data were also prevalent, particularly the mention of COVID-19 and being in an unprecedented time. It is worth noting that all motivations mentioned previously are other oriented (Chin et al. 2016). But we also identified some self-oriented motivations albeit relatively less common, namely interest and continuity. Interest can be interpreted as tangible rewards, such as using the survey as an opportunity to monitor their own mental health or receiving feedback on research findings from the study team (via newsletters). But it may also include intangible rewards where people simply find the study relevant and interesting (Han et al. 2009). Motivations related to continuity are in line with the commitment theory, which explains why people engage in consistent lines of activities, such as follow-up surveys (Keusch 2015).

Overall, these retention strategies are all broadly to do with “creating a project community” as defined in previous meta-analyses (Teague et al. 2018). This was a feature that CSS particularly focused on (e.g., emphasizing the benefits of the study, writing new emails with every wave encouraging people to stay involved, sharing study and results via newsletters, study website, and media).

### Predictors of Attrition and Re-engagement

Despite high retention rates overall, the CSS was not exempt from attrition. Not surprisingly, attrition was systematically related to individual characteristics, such as gender, age socioeconomic position, living with children and so forth. These findings are largely consistent with previous research before COVID-19 (Behr et al. 2005; Uhrig 2008; Watson and Wooden 2009). Previous studies also found evidence for the association between health and attrition (Beller et al. 2022). We found evidence for the association of attrition only with mental health, but not with physical health. This might be explained by the results from the free-text data on challenges where mental health was reported as one of the common challenges. However, none of the top features was related to physical health, except for the feature “covid,” which was used to describe challenges due to COVID-19 symptoms by a small number of participants.

In addition to individual characteristics, invitation timing was related to survey attrition. More specifically, people who received survey invites on Monday to Thursday were at a higher risk to attrite. In the free-text data, some participants reported that if the survey invites arrived at an inconvenient time, it was challenging for them to remember to complete the survey afterwards. Although people’s preferences may vary, our findings suggest that weekends might work better for participants, regardless of employment status. This finding is at odds with an early experimental study on survey response in Sweden, which found that weekend days were associated with lower response compared to weekdays (Lindgren et al. 2018), calling for further investigation. Moreover, we also found some evidence that attrition was associated with country of residence. People living in Scotland and Northern Ireland were at a higher risk of attrition compared to England. This could be due to differences in societal-level factors, but it might also be related to the questionnaire design. For example, in the free-text question on challenges, some participants commented that some questions on central government were less relevant for people living in other devolved countries. However, this would not explain why people living in Wales had a lower risk to attrite. Future work is encouraged to inspect the country difference in greater detail.

Similar to attrition, we found that most of the individual characteristics were also related to re-engagement. However, unlike attrition, we found no evidence for the association with re-engagement for other variables (e.g., care giving, area of living, country, mental health). Moreover, taking advantage of group randomization, we were able to assess the impact of social context on re-engagement, finding that the group that received the survey invite in the third week (September 7–13, 2020) were less likely to rejoin the study. A possible explanation is that in this week, the COVID-19 restrictions started to tighten post the first national lockdown, and the “rule-of-six” was introduced in response to the increase of COVID-19 cases and deaths. As shown in previous studies (Aknin et al. 2022; Bu et al. 2023), greater pandemic intensity and policy stringency were associated with poor mental wellbeing, which arguably might influence survey participation. Interestingly, no difference was found for the fourth group who received the survey invitation when the rule came into effect (September 14, 2020). This is consistent with earlier research, showing that much of the psychological toll was experienced in the anticipation of policy restrictions (Fancourt et al. 2021).

### Learnings for Future Studies

There are some important lessons to be learned from these findings. First, when carrying out online panel studies, it is important to keep a record of attempted sample for each wave to accurately estimate retention/attrition. Second, it is worthwhile to contact dropout participants, as this can be effective in maintaining the study sample size. However, the self-selection bias due to attrition might be reinforced when dropout participants rejoined the study given a similar set of factors were related to both attrition and re-engagement. Third, our findings suggested that despite being closely linked, re-engagement might have some distinct decision processes compared to attrition, which calls for further theoretical and empirical research.

Given attrition is likely to lead to bias and lower precision, it is important to minimize attrition during the data collection phrase. A number of techniques have been proposed in the literature, such as reducing follow-up frequency, as well as the length and complexity of the questionnaire, increasing the number and diversity of contact attempts; and so forth (Laurie 2008; Lynn 2017). In the case of CSS, we addressed this through our citizen science design ethos: We tried to keep in touch with participants via email and newsletters, including study updates, summaries of key findings, and a thank-you video released on the one-year anniversary of the study, which might have helped encourage continued engagement to some extent. Reports on the motivations to participate about helping, feeling the research was important, and wanting to contribute suggest that this citizen science approach was valuable in helping participants feel like peer researchers in the study. Thus, co-production strategies are recommended in similar future studies.

When attrition does inevitably occur, a number of techniques post-data collection can be used to reduce the statistical impact of attrition, including weight adjustment, multiple imputation, and full information maximum likelihood (Asendorpf et al. 2014; Cumming and Goldstein 2016; Lynn 2017). The CSS data comes with a cross-sectional weight. Longitudinal weights are not provided due to the large number of follow-ups and the fact that participants might join the study at different time points (mostly March–May 2020). But we encourage data users to develop their own weights, especially for studies that use a sub-set of the sample or focus on a specific period of time.

### Limitations

This study has a number of strengths, including its large sample size, unique study designs, high quality and rich data over different stages of the COVID-19 pandemic, and so forth. But it comes with limitations. First, the CSS used convenience sampling, which may influence the generalizability of our findings despite of weighting due to omitted variable biases. Second, although we were able to identify and exclude noncontacts due to undeliverable email addresses, we could not examine other sources of attrition (e.g., refusal, mortality). Future studies are therefore recommended to use innovative approaches to collect information on sources of attrition. Third, the free-text data have provided valuable information on motivations and challenges that help us interpret our findings on retention/attrition and re-engagement. But it should be acknowledged that the free-text data were collected at the end of the study (March/April 2022), which means that participants who dropped out the study by then did not have the opportunity to respond to these questions. And no information was available for those who never participated in the study.

## Conclusions

This study examined factors that are associated with attrition and re-engagement in the CSS, including social context, survey design, and individual characteristics. It contributes to our understanding of participation in online panel studies. It also adds to the literature regarding motivations and challenges of survey participation during a global health crisis. Our findings are of particular importance for researchers who use data from the CSS or other studies with a similar design, and shed light on the design, implementation, and data analysis of other online panel surveys.

## Supplemental Material

Supplemental Material - Online Survey Retention and Re-Engagement: Learning From the COVID-19 Social Study Supplemental Material for Online Survey Retention and Re-engagement: Learning From the COVID-19 Social Study by Feifei Bu, Alexandru Cernat, Andrew Steptoe, and Daisy Fancourt in Field Methods.
