# Supplementary material for: Online Survey Retention and Re-engagement: Learning from the COVID-19 Social Study
Source: Field methods. 2025 Jan 8;37(3):244–59. doi: 10.1177/1525822X241289870 (PMC12398353; doi:10.1177/1525822X241289870)
Supplement: Supplemental Material - Online Survey Retention and Re-Engagement: Learning From the COVID-19 Social Study [file sj-pdf-1-fmx-10.1177_1525822X241289870.pdf]

Supplementary Material

Table S1 Sample characteristics comparing to the UK adult population estimates

		Raw data	Weighted data*	Population estimates†
**Gender**	Men	25.1%	49.3%	49.4%
	Women	74.9%	50.7%	50.6%
**Ethnicity**	White	93.9%	87.2%	87.2%
	Ethnic minority	6.1%	12.8%	12.8%
**Age groups**	18–29	10.9%	19.5%	19.5%
	30–45	31.2%	26.1%	26.1%
	46–59	30.4%	24.1%	24.1%
	60+	27.5%	30.3%	30.2%
**Education**	GCSE or below (low)	15.2%	32.6%	32.7%
	A-levels or equivalent (medium)	18.2%	33.8%	33.9%
	Degree or above (high)	66.7%	33.5%	33.4%
**Country**	England	81.7%	84.3%	84.3%
	Wales	10.6%	4.7%	4.7%
	Scotland	6.6%	8.2%	8.2%
	Northern Ireland	1.2%	2.8%	2.8%
**Area**	Rural	22.6%	20.6%	15.8%
	Urban	77.4%	79.4%	84.2%
**Household income**	<30k (low)	39.2%	49.0%	--
	≥30k (high)	60.8%	51.0%	--
**Employment**	Employed	65.2%	59.6%	--
	Other	34.8%	40.4%	--
**Living status**	With children	29.5%	28.5%	--
	No children	70.5%	71.5%	--
**Carer**	Yes	25.1%	24.5%	--
	No	74.9%	75.5%	--
**Poor physical health**	Yes	38.9%	41.4%	--
	No	61.1%	58.6%	--
**Poor mental health**	Yes	19.8%	20.6%	--
	No	80.2%	79.4%	--

Notes: * Weights were generated using entropy balancing weight accounting for gender, ethnicity, age, education, and country of living. † Population estimates are from the Office for National Statistics (year 2020), except for area of living which is from World Bank (year 2020, whole population). – No national population data available

Table S2 Sample sizes and retention rates across weeks during phase 1

Week	*N*	N with any follow-up	N with adjacent follow up	Retention rate (any)	Retention rate (consecutive)
1	27,694	22,163	20,760	80.0%	75.0%
2	27,291	24,368	22,341	89.3%	81.9%
3	37,559	33,593	30,160	89.4%	80.3%
4	37,895	34,337	30,708	90.6%	81.0%
5	38,002	33,506	29,652	88.2%	78.0%
6	36,261	32,425	27,510	89.4%	75.9%
7	36,340	33,511	30,292	92.2%	83.4%
8	37,060	33,888	30,945	91.4%	83.5%
9	35,310	33,079	29,704	93.7%	84.1%
10	33,115	31,373	28,187	94.7%	85.1%
11	32,050	30,617	27,657	95.5%	86.3%
12	31,186	29,876	26,954	95.8%	86.4%
13	30,122	28,963	25,999	96.2%	86.3%
14	29,089	28,075	25,292	96.5%	86.9%
15	28,458	27,367	24,535	96.2%	86.2%
16	27,438	26,445	23,690	96.4%	86.3%
17	26,654	25,683	23,009	96.4%	86.3%
18	25,920	24,985	22,166	96.4%	85.5%
19	24,944	24,048	21,576	96.4%	86.5%
20	24,486	23,578	21,244	96.3%	86.8%
21	23,818	20,565	20,565	86.3%	86.3%
22	23,082				

Table S3 Sample sizes and retention rates across months during phases 2 and 3

Week	*N*	*N* with any follow-up	*N* with adjacent follow up	Retention rate (any)	Retention rate (consecutive)
1	39,087	36,794	32,639	94.1%	83.5%
2	33,306	32,423	30,063	97.3%	90.3%
3	30,529	29,957	27,785	98.1%	91.0%
4	28,298	27,890	26,217	98.6%	92.6%
5	26,829	26,588	25,332	99.1%	94.4%
6	25,986	25,766	24,435	99.2%	94.0%
7	25,105	24,776	23,324	98.7%	92.9%
8	35,881	33,044	28,649	92.1%	79.8%
9	29,765	28,709	25,775	96.5%	86.6%
10	26,738	26,045	23,652	97.4%	88.5%
11	24,629	24,259	22,326	98.5%	90.6%
12	23,174	22,841	21,032	98.6%	90.8%
13	21,979	21,719	20,105	98.8%	91.5%
14	21,027	20,829	19,347	99.1%	92.0%
15	20,150	19,934	14,616	98.9%	72.5%
16	15,462	15,244	14,773	98.6%	95.5%
17	32,582	28,812	26,203	88.4%	80.4%
19	32,158	23,751	23,751	73.9%	73.9%
21	29,337				

Table S4 Results from Cox regression model on attrition (phase 1, March–August 2020)

	Unweighted (*N*=62,722)		Weighted (*N*=62,722)	
	HR	95% CI	HR	95% CI
Women (vs. men)	**0.85**	**[0.84-0.87]**	**0.85**	**[0.82-0.88]**
White (vs. other)	**0.78**	**[0.75-0.81]**	**0.77**	**[0.73-0.81]**
Age 30–45 (vs. 18–29)	**0.68**	**[0.65-0.70]**	**0.67**	**[0.64-0.70]**
Age 46–59 (vs. 18–29)	**0.45**	**[0.43-0.46]**	**0.44**	**[0.42-0.46]**
Age 60+ (vs. 18–29)	**0.28**	**[0.27-0.29]**	**0.28**	**[0.27-0.30]**
A-levels or equivalent (vs GCSE or below)	**0.82**	**[0.79-0.85]**	**0.82**	**[0.79-0.86]**
Degree or above (vs GCSE or below)	**0.71**	**[0.69-0.74]**	**0.70**	**[0.67-0.72]**
Wales (vs. England)	**0.95**	**[0.91-0.98]**	**0.87**	**[0.82-0.91]**
Scotland (vs. England)	**1.07**	**[1.03-1.11]**	**1.06**	**[1.00-1.12]**
Northern Ireland (vs. England)	**1.20**	**[1.10-1.31]**	**1.34**	**[1.19-1.50]**
Urban (vs. rural)	**1.03**	**[1.00-1.05]**	1.01	[0.98-1.05]
Low household income (vs. high)	**1.06**	**[1.04-1.08]**	**1.09**	**[1.06-1.13]**
Employed (vs. not employed)	**1.19**	**[1.16-1.22]**	**1.19**	**[1.15-1.24]**
Living with children (vs. not)	**1.29**	**[1.27-1.32]**	**1.26**	**[1.22-1.30]**
Carer (vs. not)	**1.09**	**[1.06-1.11]**	**1.07**	**[1.03-1.11]**
Poor physical health (vs. good)	1.02	[0.99-1.04]	1.02	[0.99-1.06]
Poor mental health (vs. good)	**1.10**	**[1.07-1.13]**	**1.07**	**[1.03-1.11]**
Monday (vs. Sunday)	**1.21**	**[1.16-1.25]**	**1.18**	**[1.11-1.25]**
Tuesday (vs. Sunday)	**1.37**	**[1.32-1.42]**	**1.41**	**[1.34-1.48]**
Wednesday (vs. Sunday)	**1.27**	**[1.23-1.32]**	**1.26**	**[1.20-1.33]**
Thursday (vs. Sunday)	**1.18**	**[1.14-1.23]**	**1.15**	**[1.09-1.22]**
Friday (vs. Sunday)	1.02	[0.98-1.06]	1.06	[1.00-1.12]
Saturday (vs. Sunday)	1.04	[1.00-1.09]	**1.09**	**[1.03-1.16]**

Table S5 Results from logistic regression models on re-engagement (phase 2)

	Phase 2 (N=39,088)			
	Unweighted		Weighted	
	OR	95% CI	OR	95% CI
Women (vs. men)	**1.27**	**[1.20,1.33]**	**1.24**	**[1.16,1.33]**
White (vs. other)	**1.38**	**[1.26,1.51]**	**1.41**	**[1.23,1.63]**
Age 30–45 (vs. 18–29)	**1.50**	**[1.39,1.62]**	**1.62**	**[1.45,1.80]**
Age 46–59 (vs. 18–29)	**2.29**	**[2.11,2.47]**	**2.62**	**[2.34,2.92]**
Age 60+ (vs. 18–29)	**3.51**	**[3.21,3.83]**	**4.01**	**[3.54,4.54]**
A-levels or equivalent (vs. GCSE or below)	**1.31**	**[1.21,1.42]**	**1.43**	**[1.29,1.57]**
Degree or above (vs. GCSE or below)	**1.57**	**[1.47,1.68]**	**1.68**	**[1.54,1.83]**
Wales (vs. England)	1.03	[0.95,1.11]	1.06	[0.94,1.19]
Scotland (vs. England)	**0.90**	**[0.83,0.98]**	0.88	[0.77,1.01]
Northern Ireland (vs. England)	0.93	[0.76,1.12]	0.83	[0.62,1.11]
Urban (vs. rural)	0.99	[0.94,1.04]	1.00	[0.92,1.08]
Low household income (vs. high)	**0.85**	**[0.81,0.90]**	**0.83**	**[0.77,0.90]**
Employed (vs. not employed)	**0.93**	**[0.88,0.99]**	**0.90**	**[0.83,0.98]**
Living with children (vs. not)	**0.88**	**[0.84,0.93]**	**0.90**	**[0.84,0.98]**
Carer (vs. not)	0.97	[0.93,1.03]	1.01	[0.94,1.10]
Poor physical health (vs. good)	1.00	[0.95,1.05]	0.94	[0.87,1.01]
Poor mental health (vs. good)	0.97	[0.92,1.03]	1.00	[0.92,1.09]
Group 2 (vs. group 1)	1.03	[0.97,1.09]	1.02	[0.92,1.12]
Group 3 (vs. group 1)	**0.93**	**[0.87,0.99]**	**0.90**	**[0.82,0.99]**
Group 4 (vs. group 1)	1.00	[0.94,1.06]	0.97	[0.88,1.06]

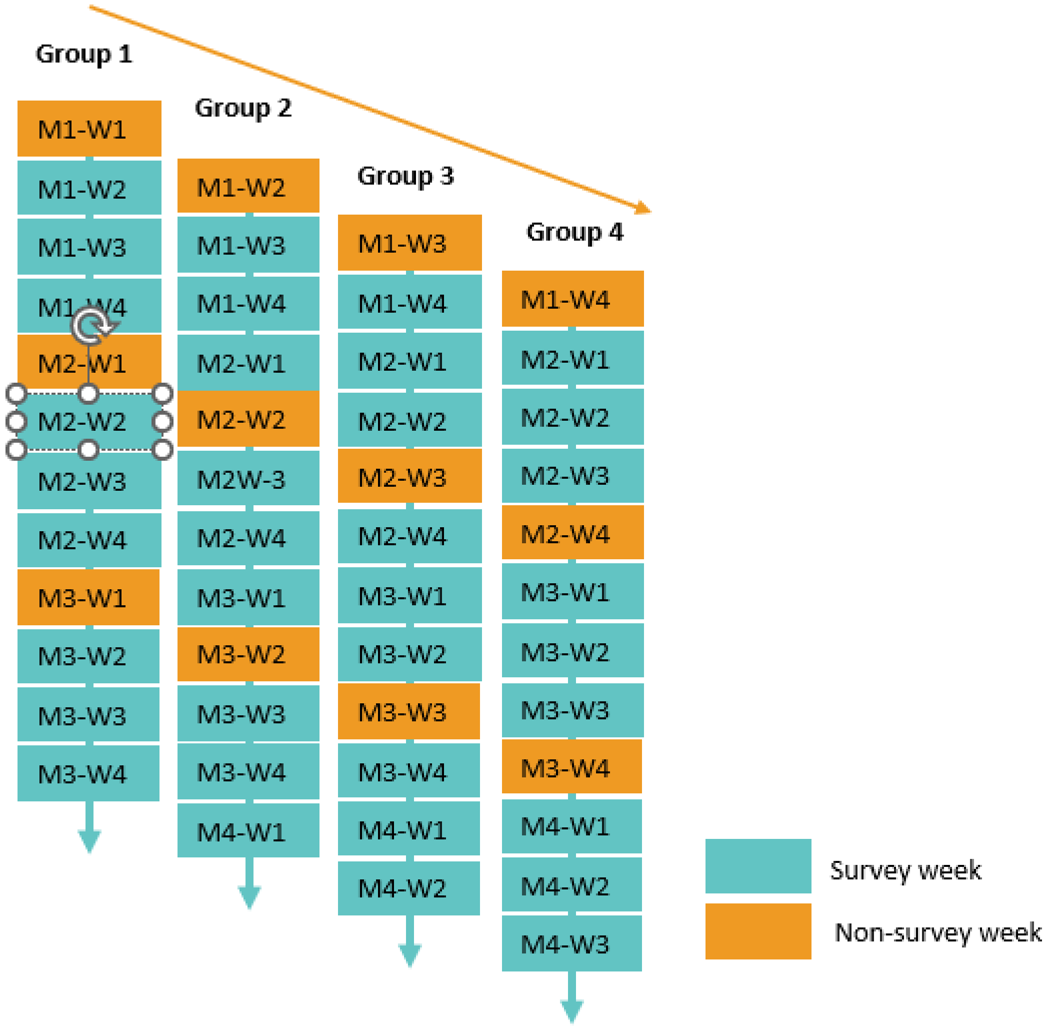

Figure S1. Randomly assigned groups in phase 2 and their survey timelines (M-month, W-week, e.g. M1-W1 the first week of the first monthly survey)

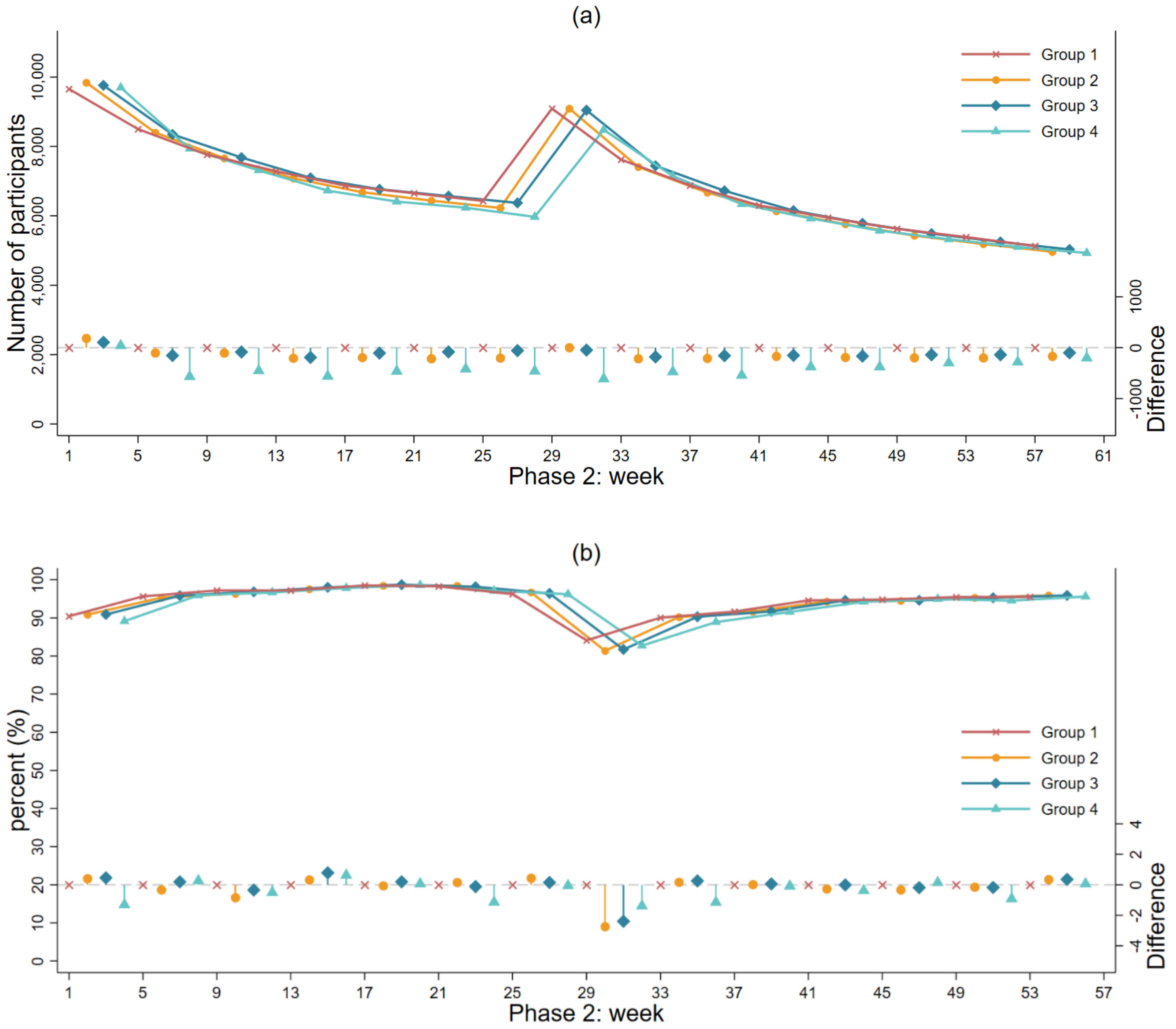

Figure S2. Difference in retention rates in phase 2 across randomly assigned groups (a) number of participants (b) Retention rate: % with any follow-up

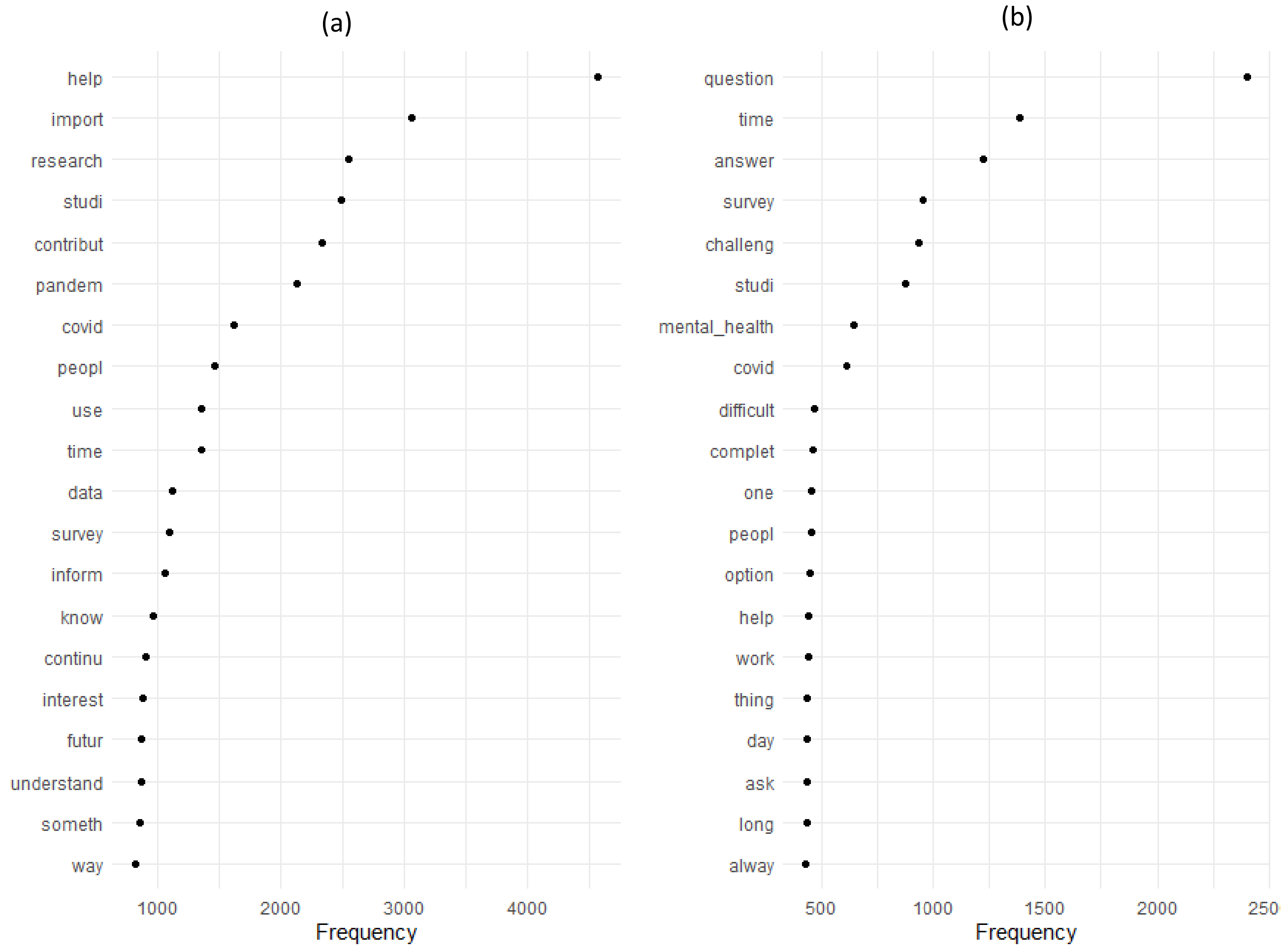

Figure S3. Top 20 features in the responses to the motivation (a) and challenge (b) free-text questions
